# Activation of Alveolar Macrophages with Interferon-γ Promotes Antioxidant Defenses via the Nrf2-ARE Pathway

**DOI:** 10.4172/2155-9899.1000365

**Published:** 2015-10-30

**Authors:** Bashar S Staitieh, Eduardo E Egea, Xian Fan, Nnamdi Azih, Wendy Neveu, David M Guidot

**Affiliations:** 1 Division of Pulmonary, Allergy & Critical Care Medicine, Emory University School of Medicine, United States; 2 Morehouse School of Medicine, 720 Westview Drive SW, Atlanta, United States; 3 Atlanta VA Medical Center, Decatur, United States

**Keywords:** Oxidative stress, Nrf2, iNOS, IL-4, Alveolar macrophage, Innate immunity, Macrophage polarization

## Abstract

Macrophage phenotype and function is dependent on the underlying microenvironment. Many diseases are accompanied by abnormal shifts in macrophage polarization state that limit the ability of the cells to become innate immune effectors. Previous work in the field suggests that chronic alcohol ingestion, which is associated with a shift away from innate immune effector macrophages, is also associated with a deficient response to oxidative stress. We therefore hypothesized that the optimal response to oxidative stress was dependent on the ability of the macrophage to become an innate immune effector cell. To investigate this hypothesis, we first confirmed that we could reproducibly polarize NR8383 cells (a rat alveolar macrophage cell line) into the prototypical M1 and M2 states (using IFN-γ and IL-4, respectively). We then tested the polarized cells for their ability to scavenge reactive oxygen species generated by glucose oxidase (GOX) using the Amplex red assay and found that IFN-γ-polarized cells had greater scavenging capacity. To elucidate the mechanism of the enhanced response to oxidative stress, we then assessed key components of the anti-oxidant response; specifically, nuclear factor (erythroid-derived 2)-like 2 (Nrf2), the master transcription factor responsible for the cellular response to oxidative stress, and one of its downstream effectors, glutamate-cysteine ligase catalytic subunit (GCLC). We found that both proteins were significantly upregulated in the IFN-γ-polarized cells. To confirm that Nrf2 is an integral component of this improved anti-oxidant response, we transfected IFN-γ-polarized cells with either silencing RNA to Nrf2 or control silencing RNA and found that hydrogen peroxide scavenging was significantly impaired in the si-Nrf2-treated cells. Further, transfecting untreated cells with si-Nrf2 polarized them toward the M2 phenotype in the absence of IL-4, suggesting a mechanistic role for Nrf2 in macrophage polarization. We then confirmed several of our key experiments in primary rat alveolar macrophages cells. Taken together, these findings suggest that the M1 polarization state is necessary for the optimal response to oxidative stress in the macrophage, and that this response is mediated through Nrf2 and its downstream effectors.

## Introduction

Macrophages dynamically alter their phenotype and function in response to the surrounding microenvironment [1]. Although the nomenclature for these changes in polarization is controversial and many different classification schemes exist [2], the spectrum of polarization states in the macrophage is generally thought to range between the innate immune effector cell (commonly referred to as the M1, or classically-activated macrophage) and the wound-healing cell (often referred to as the M2, or alternatively-activated macrophage). Certain pathological conditions, including chronic obstructive pulmonary disease (COPD) [3] and human immunodeficiency virus (HIV) infection, limit macrophage plasticity and thereby restrict their functional capabilities [4]. Previous studies on chronic alcohol ingestion in both human subjects and animal models has characterized two key defects in the lung-specific alveolar macrophages; specifically, these macrophages appear to be skewed toward the M2 polarization state [5] and lack the ability to respond efficiently to oxidative stress [6] and bacterial infections [7]. The deficient response to oxidative stress can be linked to impaired signaling through nuclear factor (erythroid-derived 2)-like 2 (Nrf2), the master transcription factor responsible for the cellular response to oxidative stress [8]. Activation of Nrf2 programmatically activates hundreds of genes by binding to a consensus sequence known as the anti-oxidant response element (ARE) in the promoter regions of Nrf2-responsive genes [9].

Based on the paired observations of the persistent skewing toward the M2 state and the associated defect in Nrf2 signaling caused by chronic alcohol exposure, we hypothesized that Nrf2 may require M1 polarization in order to function optimally. When activated by an innate immune stress, the M1 macrophage not only generates reactive oxygen species (ROS) including hydrogen peroxide and superoxide anion in order to kill pathogens, but it must do so in the context of acute inflammatory stress in an already highly oxidizing environment within the lower airways. Therefore, the M1 polarized macrophage must also be able to withstand significant oxidative stress [10]. In this context it is reasonable to predict that M1 polarized macrophages would up-regulate their anti-oxidant capacity, and that this should be reflected by a relative increase in Nrf2 and Nrf2-ARE-dependent production of anti-oxidants. Therefore, we determined the relative capacities of the Nrf2-ARE axis in macrophages that were polarized toward either the M1 or the M2 state.

## Materials and Methods

### Cell culture

A rat alveolar macrophage cell line (NR8383; ATCC, Manassas, VA, USA) was cultured in F12K (ATCC) with 10% FBS (Atlanta Biologicals, Lawrenceville, GA, USA) with penicillin, streptomycin, and amphotericin B (Sigma-Aldrich, St. Louis, MO, USA) at 37°C in 5% CO_2_. Plating of cells with cytokine treatments was done with F12K +5% FBS. Primary alveolar macrophages were obtained by lavage from 6 week-old Fischer rats and cultured in F12K (Mediatech, Manassas, VA)+10% FBS with treatments as described below. All procedures were approved by the Institutional Animal Care and Use Committee at the Atlanta Veterans Affairs and Emory University.

### Cytokine treatments

NR8383 cells (250,000 cells/well in a 24-well plate) were treated with either 30 ng/ml of rat interferon-γ (IFN- γ; R&D Systems, Minneapolis, MN, USA) or 20 ng/ml rat interleukin-4 (IL-4; PeproTech, Rocky Hill, NJ, USA) for 72 hours. Primary rat alveolar macrophages were treated with the same concentration of cytokines for 48 hours.

### RNA isolation and RT-PCR

Total RNA was extracted using the Qiagen RNeasy Mini kit (Valencia, CA, USA). Reverse transcription and real-time PCR (Bio- Rad Lab, Hercules, CA, USA) were performed as described previously [11] using primer pairs shown in Table 1. PCR products were normalized to Quantum RNA Universal 18S Internal Standard (Life Technologies, Grand Island, NY, USA) from the same RT sample.

### Amplex red assay

Amplex red hydrogen peroxide/peroxidase assay kit (Life Technologies) was used to assess hydrogen peroxide concentration according to the Life Technologies protocol. Briefly, after treatment, cells were plated in Kreb-Ringer Phosphate Buffer with glucose oxidase at 5 mU/ml (GOX; Sigma-Aldrich) for 2 hours prior to re-plating with Kreb-Ringer Phosphate Buffer, Amplex red reagent, and GOX 5 mU/ml for another 2 hours. Media from each well was then tested for relative H_2_O_2_ concentration by spectrophotometer (Synergy H1, Biotek, Winooski, VT). Scavenging of hydrogen peroxide was calculated by subtracting the Amplex red signal of a cell free well from the signal generated by treated samples.

### Flow cytometry

NR8383 cells plated in 24 well plates and treated with IFN-γ or IL-4 for 72 hours. Cells were then detached with Versene (Life Technologies), fixed with 4% paraformaldehyde, washed with PBS, and incubated with FcγR blocking mAb (BD Biosciences, San Jose, CA, USA). Cell membrane permeabilization for intracellular staining was achieved with Permwash solution (BD). Cells were stained with the following antibodies: anti-Nrf2 mouse monoclonal antibody (Abcam, Cambridge, MA, USA), anti-GCLC rabbit polyclonal antibody (Abcam), anti-mouse Pacific Blue (Life Technologies), and anti-rabbit Alexa Fluor 555 (Life Technologies). Data was acquired with an LSR-II flow cytometer in conjunction with FACSDiva software (BD). Mean fluorescence intensities (MFI) were obtained with FlowJo (FlowJo LLC, Ashland, OR, USA).

### Nrf2 silencing RNA

Nrf2 Stealth Select RNAi (set of 3 siRNA) were obtained from Life Technologies (Nfe2l2, oligo ID: RSS343557, RSS343558, RSS343559). All three siRNA for Nrf2 were tested for previous experiments [12] and RSS343557 was more effective and chosen for this study. NR8383 cells (250,000 cells/well in a 24 well plate) were plated in OptiMEM (Life Technologies) and transfected with 10 nM of stealth RNAi for rat Nrf2 or negative control using the Lipofectamine 3000 transfection reagent (Life Technologies). Amplex red assay or RT-PCR were then conducted as outlined above.

### Statistical analysis

Student’s t-test was used for single comparisons (Microsoft Excel or GraphPad Prism for flow cytometry). Data are presented as mean ± SEM. Significance was accepted at p<0.05.

## Results

### Interferon-gamma and interleukin-4 polarize macrophages into the M1 and M2 states, respectively

**Figure 1:**
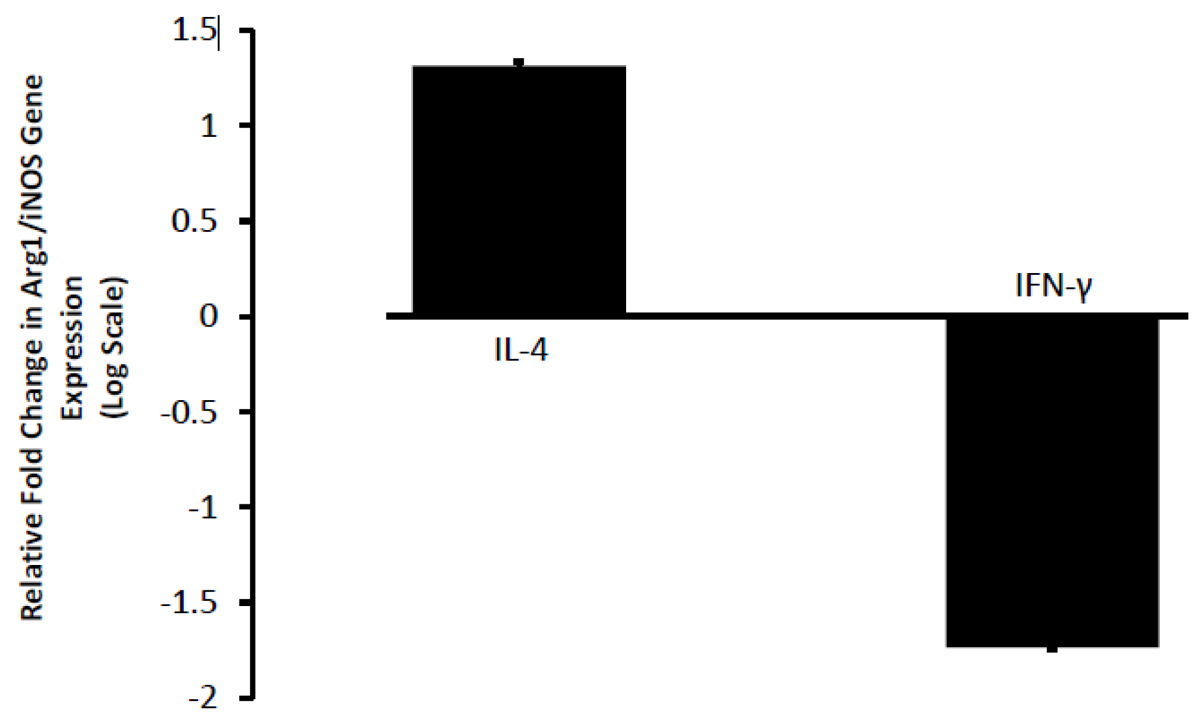
Treatment of a rat alveolar macrophage cell line with IFN- γ or IL-4 polarizes them into the M1 or M2 state, respectively NR8383 cells were treated with either IFN-γ or IL-4 for 72 hours. Gene expression of Arg1 and iNOS were quantified by qPCR. Cells treated with IFN-γ demonstrated a marked decrease in the ratio of Arg1 to iNOS expression, suggesting a shifting of the population toward the M1 activation state. Cells treated with IL-4 had a marked increase in the ratio of Arg1 to iNOS ratio, suggesting a shift of the population to the M2 state. Data presented as a ratio of the fold change on a logarithmic scale (mean ± SEM, n=4).

We used a rat alveolar macrophage cell line (NR8383 cells) and confirmed that the concentration and duration of cytokine treatments selected shifted the cells’ polarization state. To shift cells to the M1 activation state, we used interferon-gamma (IFN-γ), the canonical M1 cytokine. For the M2 state, we used interleukin-4 (IL-4), the prototypical M2 cytokine. As shown in Figure 1, IL-4 treatment markedly increased the ratio of Arginase-1 gene expression (Arg1, a marker associated with the M2 state) and inducible nitric oxide synthase gene expression (iNOS, a key marker of the M1 state), reflecting a marked shift of the population to the M2 state by qPCR. In contrast, IFN-γ treatment dramatically reduced the ratio of Arg1 and iNOS, reflecting a shift of the population toward the M1 state. Relative Arg1/iNOS ratios are presented on a log scale for clarity. Of note, survival in the two populations was not significantly different (68% ± 2% for IL-4 and 60% ± 5% for IFN-γ, p=0.18).

### Macrophages in the M1 activation state are more efficient at reducing levels of oxidative stress

**Figure 2:**
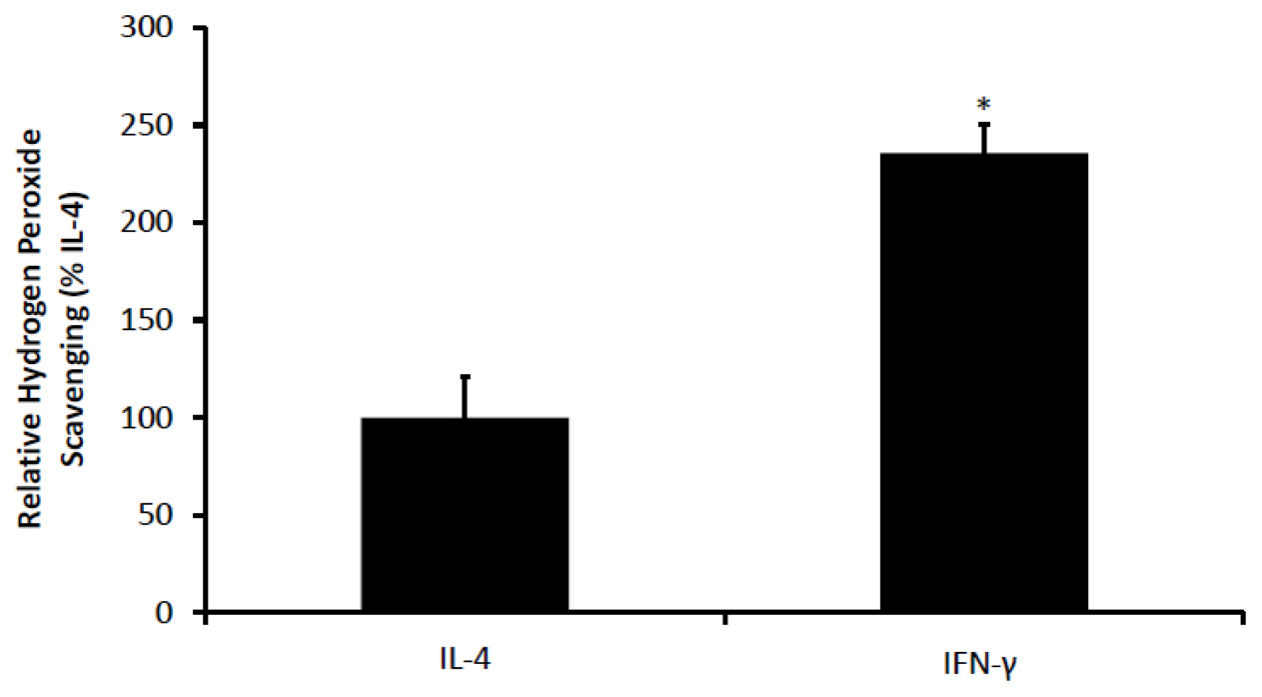
Macrophages polarized to the M1 state with IFN-γ are better at hydrogen peroxide scavenging than IL-4 treated M2 macrophages NR8383 cells were treated with IFN-γ as above. They were then treated with glucose oxidase (GOX) 5 mU/ml to produce a steady level of hydrogen peroxide (H2O2). After four hours, relative H2O2 scavenging was assessed by Amplex Red Assay by subtracting the MFI for the treated samples from a cell-free sample treated with GOX in parallel. Cells polarized to the M1 state showed a significant improvement H_2_O_2_ scavenging. Data presented as mean ± SEM, n=4. *p<0.05 compared with IL-4 treated cells.

After confirming that we could reliably polarize macrophages into the M1 and M2 states, we next exposed polarized macrophages to glucose oxidase, an enzyme that generates steady levels of hydrogen peroxide [13] and assessed the relative abilities of the M1 vs. M2 macrophages to respond to the exogenous oxidative stress and scavenge hydrogen peroxide as quantified by the Amplex red assay. As shown in Figure 2, macrophages polarized with IFN-γ toward the M1 state were significantly better at scavenging hydrogen peroxide compared to macrophages polarized with IL-4 toward the M2 state (relative scavenging activities of 236% ± 15% vs. 100% ± 21%, p<0.05).

### Nrf2-ARE activity is enhanced in the M1 state

**Figure 3:**
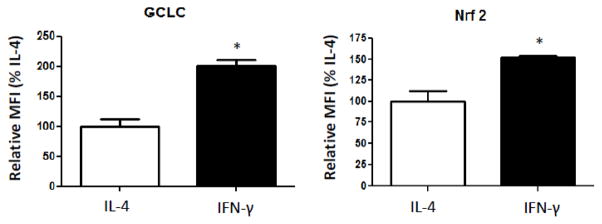
Nrf2-ARE activity is enhanced in cells polarized with IFN- γ NR8383 cells (1 million/well) were treated with IFN-γ as above. Seventy-two hours later, protein expression of Nrf2 and its downstream effector GCLC were significantly increased in IFN-γ polarized cells as assessed by flow cytometry. Data presented as mean ± SEM, n=3. *p<0.05 compared with IL-4 treated cells. MFI=Mean fluorescence intensity.

Based on our determination that a shift toward the M2 state is accompanied by a decrease in anti-oxidant defenses, we hypothesized that the improvement in ROS scavenging in the M1 state is due to enhancement of the Nrf2-ARE axis, the key effector in the cellular response to oxidative stress. Therefore, we compared the relative protein expression Nrf2 and a downstream Nrf2-ARE-dependent protein, glutamate-cysteine ligase, catalytic subunit (GCLC), a key enzyme in the synthesis of glutathione. As shown in Figure 3, macrophages polarized toward the M1 state with IFN-γ had significantly higher expression of both Nrf2 and GCLC compared to macrophages polarized toward the M2 state with IL-4 (152% ± 2.4% vs. 100% ± 12.5% for Nrf2 and 202% ± 8.9% vs. 100% ± 13.2% for GCLC, p<0.05 for both experiments).

### Enhancement of the anti-oxidant response in IFN-γ polarized cells is dependent on Nrf2

**Figure 4:**
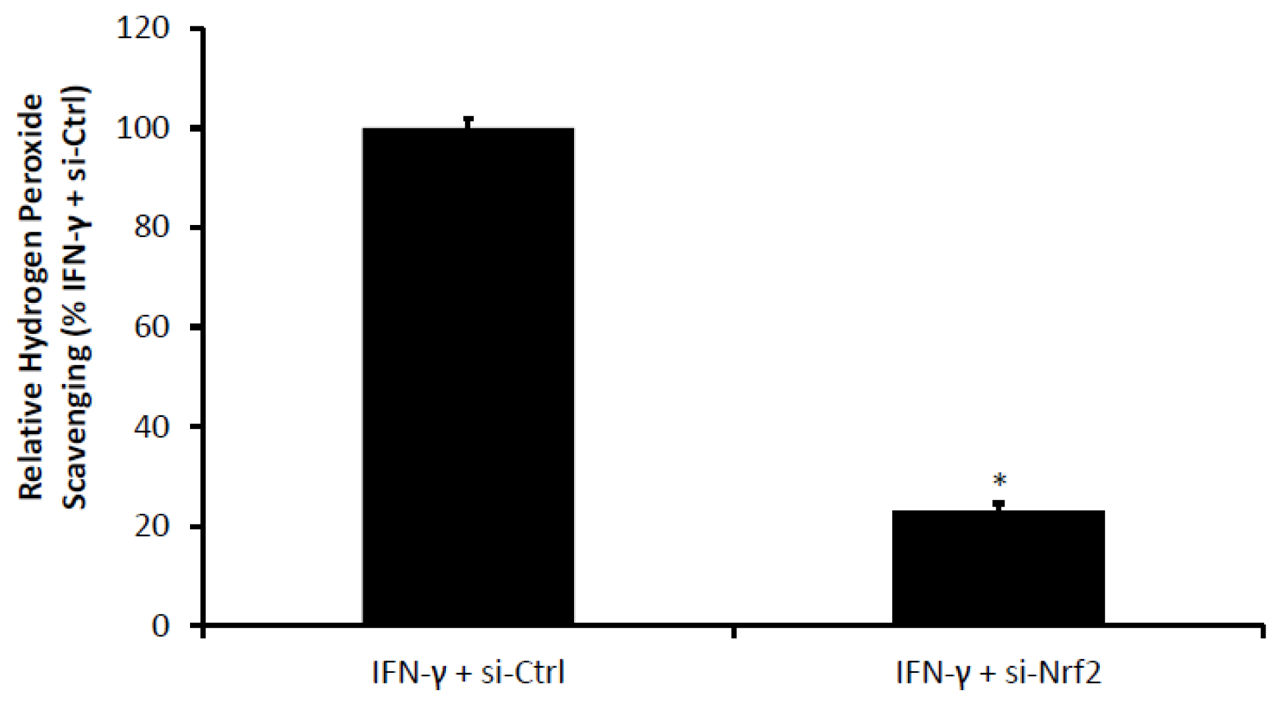
Silencing Nrf2 RNA abrogates the enhanced response to oxidative stress seen in IFN-γ treated cells NR8383 cells were polarized with either IFN-γ for 48 hours. The cells were then transfected with either silencing RNA to Nrf2 (si-Nrf2) or with a control (scrambled) silencing RNA (si-Ctrl). After transfection, cells were again plated in media containing IFN-γ. Twenty-four hours post-transfection (seventy-two hours after polarization), cells were treated with GOX (5 mU/ml). After four hours, remaining H_2_O_2_ concentration was assessed by Amplex Red Assay. Cells transfected with si-Nrf2 were significantly worse at scavenging hydrogen peroxide when compared to those transfected with si-Ctrl.

After determining that Nrf2 protein expression and signaling activity (as reflected by GCLC protein expression) is enhanced in cells polarized to the M1 state with IFN-γ, we next sought to determine whether interfering with Nrf2 signaling could abrogate the enhanced scavenging of hydrogen peroxide in the macrophages polarized by IFN-γ. We therefore transfected the IFN-γ polarized macrophages with silencing RNA to Nrf2 (si-Nrf2) and compared their hydrogen peroxide scavenging ability to IFN-γ polarized macrophages transfected with control silencing RNA (si-Ctrl). Our si-Nrf2 protocol was previously found to result in knockdown of Nrf2 RNA to approximately 60% of control levels [12]. As shown in Figure 4, IFN-γ polarized macrophages transfected with si-Nrf2 were significantly worse at hydrogen peroxide scavenging as quantified by the Amplex red assay (23% ± 1.4% vs. 100% ± 1.9% for cells transfected with si- Ctrl), indicating that the previously identified increase scavenging in the IFN-γ polarized macrophages (Figure 2) is dependent on Nrf2 and Nrf2-ARE signaling.

### Silencing Nrf2 skews macrophages toward the M2 phenotype

**Figure 5:**
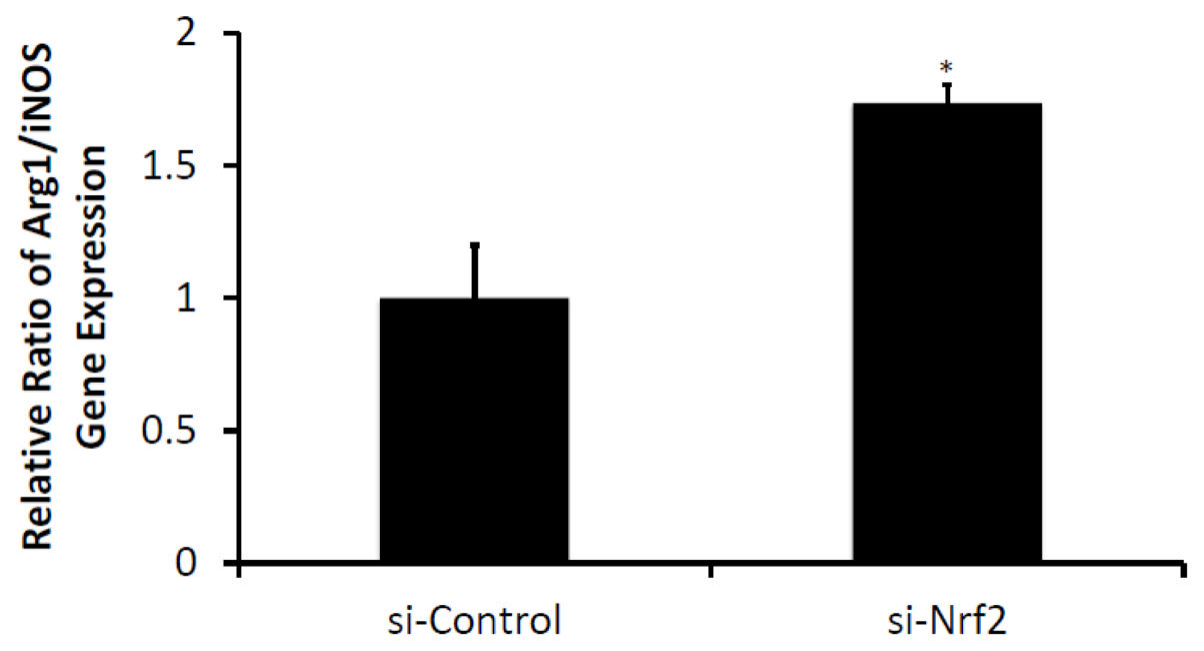
Silencing Nrf2 RNA skews macrophages toward the M2 phenotype NR8383 cells were transfected with either silencing RNA to Nrf2 (si-Nrf2) or with a control (scrambled) silencing RNA (si-Ctrl). Twenty-four hours after transfection, gene expression of Arg1 and iNOS were assessed by RT-PCR. Cells transfected with si- Nrf2 were significantly skewed toward the M2 phenotype, as shown by an elevated Arg1/iNOS ratio. Data presented as a ratio of fold change, mean ± SEM, n=5, *p<0.05.

Next, in an effort to follow-up on our recent finding that Nrf2 regulates PU.1 [12] (itself an important regulator of macrophage polarization through its intracellular transduction of GM-CSF signaling) [1], we transfected untreated cells with si-Nrf2 to determine whether it can affect macrophage polarization state. Although the effect is less pronounced than that of IL-4, as shown in Figure 5, si- Nrf2 transfection does result in a significant increase of the Arg1/iNOS ratio, reflecting a skewing of the macrophage population toward the M2 state.

### Primary rat alveolar macrophages demonstrate similar enhancements to anti-oxidant defenses when activated with IFN-γ

**Figure 6:**
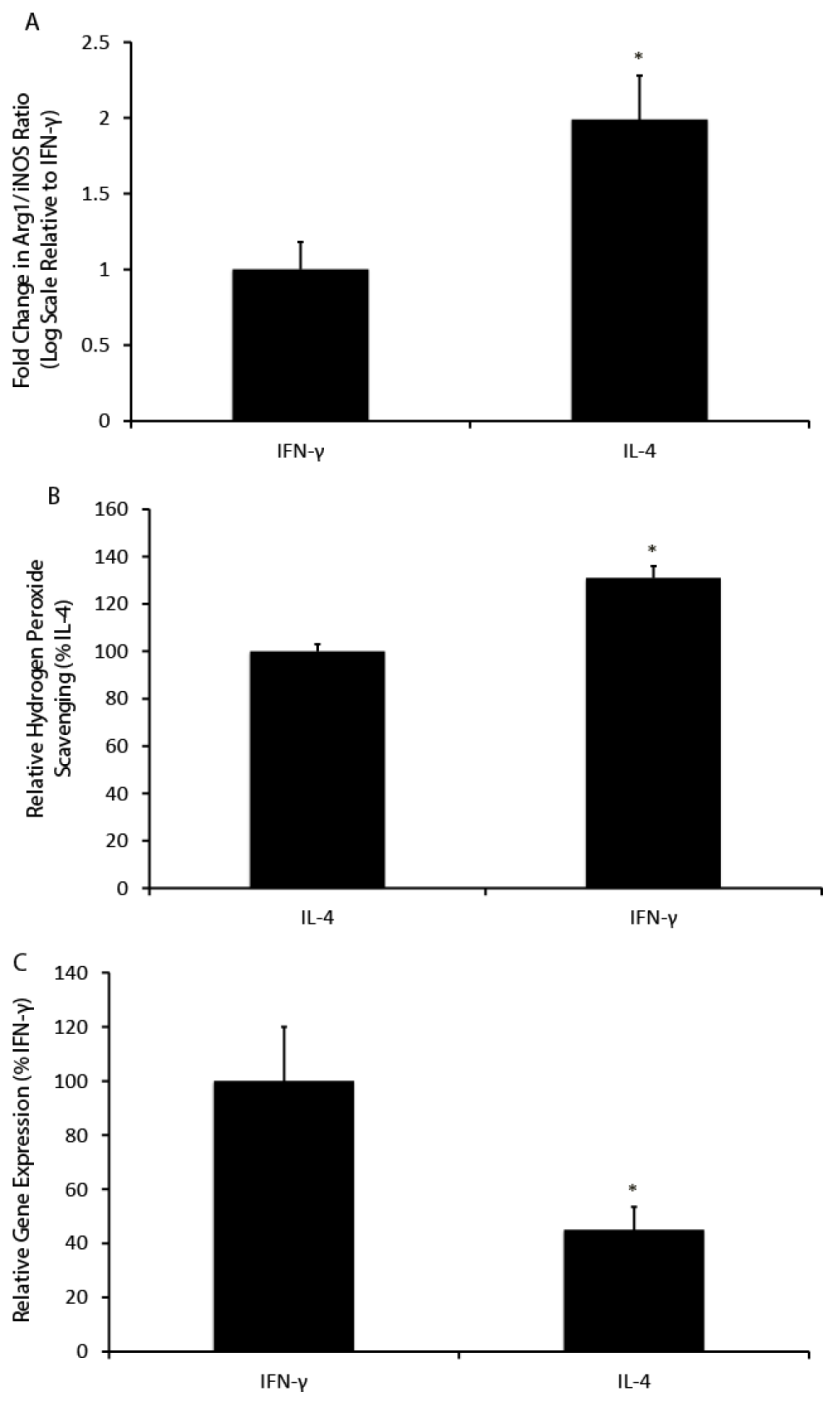
Activation of primary rat alveolar macrophages with IFN- γ and IL-4 replicates the effects seen in NR8383 cells (**A**) Forty-eight hours after treatment with IFN-γ and IL-4, gene expression of Arg1 and iNOS was assessed in primary rat alveolar macrophages. Relative to the IFN-γ-treated cells, IL-4-treated cells were significantly skewed toward the M2 state, as reflected by an elevated Arg1/iNOS ratio. Data presented as relative fold change on log scale, mean ± SEM, n=4. *p<0.05. (**B**) Primary rat alveolar macrophages treated with IFN-γ or IL-4 were challenged with 5 mU/ml GOX. After four hours, remaining H_2_O_2_ concentration was assessed by Amplex Red Assay. Cells treated with IFN-γ were significantly better at scavenging hydrogen peroxide. Data presented as relative scavenging activities compared to IL-4-treated cells, mean ± SEM, n=4, *p<0.05. (**C**) After forty-eight hours of treatment with IFN-γ or IL-4, gene expression of GCLC was assessed in the two groups as a surrogate for Nrf2 nuclear binding. Expression was significantly higher in cells treated with IFN-γ. Data presented as relative gene expressions, mean ± SEM, n=5, *p<0.05.

We next sought to confirm our findings from the cell line in primary rat alveolar macrophages. As shown in Figure 6.A, treatment with IFN-γ and IL-4 results in a significant skewing of the macrophage populations to the M1 and M2 state, respectively. Survival in the two populations was not significantly different (79% ± 4% with IFN-γ and 76% ± 8% with IL-4, p=0.72). We then challenged the treated cells with 5 mU/ml GOX, as we did in the cell line, and again found a marked increase in hydrogen peroxide scavenging in the IFN-γ-activated cells, as shown in Figure 6B (relative scavenging activities of 100% ± 3% in IL-4 treated cells vs. 131% ± 5% in IFN-γ treated cells, p<0.05). Finally, we assessed gene expression of GCLC to determine levels of Nrf2 nuclear binding after the two cytokine treatments and, as shown in Figure 6.C, confirmed that GCLC expression was significantly lower in the IL-4-treated system (100% ± 20% vs. 45% ± 8.5%, p<0.05).

## Discussion

To investigate the consequences of abnormal macrophage polarization on the anti-oxidant response, we first treated an alveolar macrophage cell line (NR8383) with canonical cytokines to polarize them toward the M1 and M2 states (IFN-γ and IL-4, respectively). After determining that these treatments polarized the macrophages effectively, we then used an Amplex red assay to determine that M1 macrophages were better able to scavenge exogenous hydrogen peroxide generated by glucose oxidase. To determine the potential mechanism(s) of this improved response, we assessed the Nrf2/ARE pathway, the key mediator of cellular defenses against oxidative stress, and identified that the M1 polarized macrophages express more Nrf2 protein and more of the Nrf2-dependent GCLC protein. To test whether the improvement in anti-oxidant responses in the M1 macrophage was in fact dependent on Nrf2 function, we used transfection-mediated silencing of Nrf2 RNA to decrease its activity [12] and found that si-Nrf2 treatment significantly decreased the hydrogen peroxide scavenging capacity of the IFN-γ polarized M1 macrophages. Interestingly, si-Nrf2 treatment alone was able to skew macrophages toward the M2 phenotype even in the absence of IL-4 stimulation. Finally, we replicated many of our key findings in primary rat alveolar macrophages to lend credence to the generalizability of our findings beyond the cell line. Taken together, these findings suggest that the impairment in anti-oxidant responses within the lower airways observed in patients with diseases such as chronic alcohol abuse may result from the skewing of their alveolar macrophages toward the M2 state and consequent impairment of the Nrf2-ARE axis. This finding helps explain why patients with impaired macrophage polarization not only have defects in innate immune function but also impairments in anti-oxidant defenses.

While we believe the evidence here is novel and provocative, we realize that these findings need to be confirmed in animal models of ‘polarization-skewing’ diseases to confirm their biological relevance in vivo. However, by placing anti-oxidant responses in the orbit of macrophage polarization state, our studies suggest new potential therapeutic options for patients with impaired anti-oxidant responses and altered macrophage polarization. Further, our finding that Nrf2 appears to have a significant effect on macrophage polarization provides a novel application of our recent finding that Nrf2 regulates PU.1 [12], a key regulator of macrophage polarization and innate immune function.

As noted earlier, there remains substantial controversy in the literature regarding the nomenclature and relevance of macrophage polarization. Many different classification schemes exist and there is a rapidly evolving opinion within the scientific community that the current M1 vs. M2 paradigm of macrophage polarization is not complex enough to account for the diversity of macrophage populations in vivo [14,15]. However, in the context of specific disease states that chronically alter polarization, there is a clear utility in understanding the relationship between phenotype and function. In terms of the specific classification scheme, we recognize that there is a spectrum between M1 and M2 that requires a variety of phenotypic markers to fully describe the macrophages in a specific context (such as the alveolar macrophage within the ‘alcoholic lung’). In that regard, thus study provides further evidence that assessing the relative expression of iNOS and Arg1, both of which are strongly associated with their respective phenotypes [1], provides a means of characterizing the relative polarization of the population of alveolar macrophages within a given pathological condition that correlates with both their innate immune function and their ability to withstand oxidative stress. However, we recognize that such analyses assess the mean polarization state and that there are likely sub-populations of macrophages that are variably polarized. In this context, it will be critical in future studies to assess the relative distribution of these sub-populations along the polarization spectrum with techniques such as flow cytometry with a larger panel of phenotypic markers. Despite these limitations in the current study we were able to manipulate the overall polarization state of these macrophages using the prototypical cytokines IFN-γ and IL-4 and identify fundamental differences in anti-oxidant capacity between macrophages that are skewed toward an M1 or an M2 state.

These findings have important clinical relevance as prior work from our laboratory group and others has explored the role of oxidative stress in macrophage dysfunction [7,9]. In the setting of chronic alcohol ingestion, alveolar macrophages are under significant oxidative stress and their innate immune function (particularly phagocytosis) is compromised [6,7]. When the oxidative stress is dampened with exogenous treatments, innate immune functions improve and plasticity of polarization is restored [5,16]. This study extends those findings and offers a potential alternative mechanism; specifically, the compromised innate immune function and polarization plasticity previously identified in chronic alcohol ingestion may lead directly to impaired Nrf2 function. Of note, recent evidence supports a role for iNOS (or Nos2) in the release of Nrf2 from its cytosolic chaperone, Kelch-like ECH-associated protein 1 (Keap1), through nitrosylation [17]. These data accord well with prior studies that found a decrease in Nrf2 activity in Nos2 −/− mice [18] and together suggest a possible mechanism for the enhancement of Nrf2 function in the iNOS-rich M1 macrophage. In addition, our recent finding that Nrf2 regulates PU.1 in the alveolar macrophage is confirmed here as another potential mechanism by which a primary defect in Nrf2 function could prevent macrophages from achieving optimal function as innate immune cells.

In summary, we determined that M1 polarization driven by IFN- γ is associated with enhanced anti-oxidant response and capacity compared to macrophages polarized toward the M2 state by IL-4. Further, the improvement in anti-oxidant response in the M1 polarization state appears to be mediated by the Nrf2-ARE axis. Future studies on the more general relationship between macrophage activation state and oxidative stress, and how the two respond to interventions that can supplement existing anti-oxidant defenses and/or induce Nrf2-ARE signaling, will help clarify if it is possible to manipulate macrophage polarization for therapeutic benefit.

We thank Dr. Samantha Yeligar and Dr. Joshua Chandler for their assistance in optimizing assays and troubleshooting experiments, Dr. Viranuj Sueblinvong, Dr. Michael Koval, and Dr. Samuel Molina for valuable scientific discussions, and Mr. Robert Raynor and Mr. S Todd Mills for their invaluable technical support.

## Figures and Tables

**Table 1:** Primer Pairs.

Primer	Forward	Reverse
**Rat iNOS**	5′-TAGTCAACTACAAGCCCCACG-3′	5′-GTGAGGAACTGGGGGAAACC-3′
**Rat Arg1**	5′-CAAGCTGGGAATTGGCAAA-3′	5′-GGTCCAGTCCATCAACATCA-3′

## References

[1] Murray PJ, Allen JE, Biswas SK, Fisher EA, Gilroy DW (2014). Macrophage activation and polarization: nomenclature and experimental guidelines. Immunity.

[2] Chávez-Galán L, Olleros ML, Vesin D, Garcia I (2015). Much More than M1 and M2 Macrophages, There are also CD169(+) and TCR(+) Macrophages. Front Immunol.

[3] Shaykhiev RA, Krause J, Salit Y, Strulovici-Barel BG, Harvey BG (2009). Smoking-dependent reprogramming of alveolar macrophage polarization: implication for pathogenesis of chronic obstructive pulmonary disease. Journal of immunology.

[4] Cassol E, Cassetta L, Alfano M, Poli G (2010). Macrophage polarization and HIV-1 infection. J Leukoc Biol.

[5] Brown SD, Brown LA (2012). Ethanol (EtOH)-induced TGF-beta1 and reactive oxygen species production are necessary for EtOH-induced alveolar macrophage dysfunction and induction of alternative activation. Alcoholism, clinical and experimental research.

[6] Yeligar SM, Harris FL, Hart CM, Brown LA (2012). Ethanol induces oxidative stress in alveolar macrophages via upregulation of NADPH oxidases. J Immunol.

[7] Mehta AJ, Yeligar SM, Elon L, Brown LA, Guidot DM (2013). Alcoholism causes alveolar macrophage zinc deficiency and immune dysfunction. Am J Respir Crit Care Med.

[8] Deramaudt TB, Dill C, Bonay M (2013). Regulation of oxidative stress by Nrf2 in the pathophysiology of infectious diseases. Med Mal Infect.

[9] Mehta AJ, Guidot DM (2012). Alcohol abuse, the alveolar macrophage and pneumonia. Am J Med Sci.

[10] Brüne B, Dehne N, Grossmann N, Jung M, Namgaladze D (2013). Redox control of inflammation in macrophages. Antioxid Redox Signal.

[11] Fan X, Staitieh BS, Jensen JS, Mould KJ, Greenberg JA (2013). Activating the Nrf2-mediated antioxidant response element restores barrier function in the alveolar epithelium of HIV-1 transgenic rats. Am J Physiol Lung Cell Mol Physiol.

[12] Staitieh BS, Fan X, Neveu W, Guidot DM (2015). Nrf2 regulates PU.1 expression and activity in the alveolar macrophage. Am J Physiol Lung Cell Mol Physiol.

[13] Liu J, Lu L, Li A, Tang J, Wang S (2015). Simultaneous detection of hydrogen peroxide and glucose in human serum with upconversion luminescence. Biosens Bioelectron.

[14] Martinez FO, Gordon S (2014). The M1 and M2 paradigm of macrophage activation: time for reassessment. F1000Prime Rep.

[15] Hume DA (2015). The Many Alternative Faces of Macrophage Activation. Front Immunol.

[16] Mehta AJ, Joshi PC, Fan X, Brown LA, Ritzenthaler JD (2011). Zinc supplementation restores PU.1 and Nrf2 nuclear binding in alveolar macrophages and improves redox balance and bacterial clearance in the lungs of alcohol-fed rats. Alcohol Clin Exp Res.

[17] McNeill E, Crabtree MJ, Sahgal N, Patel J, Chuaiphichai S (2015). Regulation of iNOS function and cellular redox state by macrophage Gch1 reveals specific requirements for tetrahydrobiopterin in NRF2 activation. Free Radic Biol Med.

[18] Um HC, Jang JH, Kim DH, Lee C, Surh YJ (2011). Nitric oxide activates Nrf2 through S-nitrosylation of Keap1 in PC12 cells. Nitric Oxide.

